# Understanding of Benzophenone UV Absorber-Induced Damage and Apoptosis in Human Hepatoma Cells

**DOI:** 10.3390/ijms26072990

**Published:** 2025-03-25

**Authors:** Luwei Tian, Yanan Wu, Yankun Jia, Ming Guo

**Affiliations:** College of Chemistry and Materials Engineering, Zhejiang Agriculture & Forestry University, Hangzhou 311300, China; tianluwei_vip@sina.com (L.T.); wuyanan0415@163.com (Y.W.)

**Keywords:** benzophenone UV absorbers (BPs), human hepatoma cells, BAPG-chain model, biological mechanism

## Abstract

Benzophenone UV absorbers (BPs), a widely used family of organic UV absorbers (UVAs), have attracted considerable attention for their effects on organisms in recent years. Previous research has been unable to illuminate the intricate situation of BP pollution. To address this knowledge gap, we devised a BAPG-chain model that surpasses existing approaches based on biochemical detection, antioxidant defense systems, proteins, and genes to investigate the biological mechanisms of benzophenone-1 (BP-1) and benzophenone-3 (BP-3) within human hepatoma SMMC-7721 cells as model organisms. The BAPG-chain model links the cellular model, molecular level, macroscopic scale, and microscopic phenomena by adopting a global assessment mindset. Our findings indicate that BPs induce apoptosis via the excessive production of reactive oxygen species (ROS), mitochondrial and nuclear damage, and disruption of the antioxidant stress system. Notably, BPs induce apoptosis via alterations in the expression of genes and proteins associated with apoptosis in the mitochondria. Our experimental evidence sheds light on the biological effects of BPs and highlights the need for further research in this area.

## 1. Introduction

Benzophenone UV absorbers (BPs) are the most critical members of the family of organic UV absorbers (UVAs). BPs are diverse and have in common a structural element of two benzene rings linked by a carbonyl group that can absorb high-energy radiation and re-emit it at lower energy levels. Various substituents and substitution patterns have been used to create structurally more diverse groups and obtain different types of BPs.

Benzophenone-3 (BP-3) and benzophenone-1 (BP-1) are the most widely used BPs (their structures are shown in Figure S1), protecting the human body from direct exposure to harmful UV radiation and being used as stabilizers in various materials. The risk of high exposure to BPs is now a serious concern, considering their widespread presence in mediums such as water, personal care products, and plastics. BP-3 can be absorbed directly through the skin into the bloodstream, causing the concentration of BP-3 in the bloodstream to be ten-times higher than that of other UVAs [1,2]. BP-3, a lipophilic compound, can accumulate in organs like the liver, kidneys, and spleen. This leads to higher BP-3 concentrations in organs than in the bloodstream [3]. BP-3 is degraded in the organism to BP-1 [4]. It has been reported that after 14 days of exposure of adult male zebrafish to BP-3, BP-1 and BP-3 were detected in the aquatic environment and the fish [5,6]. After feeding male rats with BP-3, BP-1 and BP-3 were also detected in their blood plasma [7]. BPs are currently ubiquitous. Studies have shown that BP-1 and BP-3 are endocrine disruptors [4,6]. BP-3 interferes with Kyoto Encyclopedia of Genes and Genomes (KEGG) pathways related to growth, learning behavior, mitogen-activated protein kinase (MAPK) signaling, PI3K–Akt signaling, and insulin secretion. Notably, in the case of insulin secretion, BP-3 induces Ca^2+^ up-regulation that may damage 13 cells. Growth abnormalities and social behavior (learning behavior) KEGG pathway disturbances may have potential impacts on populations of clown anemonefish [8]. Prenatal exposure to BP-3 can lead to apoptosis and neurotoxicity and alter levels of estrogen receptors (ERs) [9]. Furthermore, BP-1 binds to the human estrogen receptor alpha (Erα) and induces conformational changes [10]. The neurotoxicity of BPs has been reported in a limited number of studies so far. BP-3 affects the nervous system by regulating calcium signaling pathways [11]. A study recently suggested that BP-1 affects zebrafish myelin development and function, causing structural and functional defects in the central nervous system [12].

**Figure 1:**
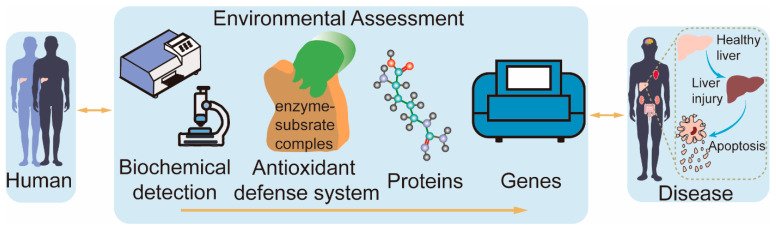
Diagram of the BAPG-chain model.

The current toxicological mechanisms of contaminants are often studied using a single assay, which does not provide a comprehensive and objective picture of the toxicological effects and mechanisms of contaminants on cells. Therefore, it is vital to find a method that reflects the toxicological mechanisms of contaminants as a whole. This study utilized heterogeneous gene–disease–trait networks [13] to develop the BAPG-chain model. The model is an advanced evaluation framework that integrates biochemical detection, antioxidant defense systems, proteins, and genes to investigate environmental toxicity mechanisms. The study aimed to investigate the toxicity of BP-3 and its primary metabolite, BP-1, in humans, and their plausible mechanisms (Figure 1). The BAPG-chain model advances the field in assessing the cytotoxic mechanisms of contaminants. Our study systematically describes the toxic effects of BP-1 and BP-3 on human cells and provides new insights into their potential mechanisms.

## 2. Results and Discussion

BPs are commonly incorporated into various commercial products [14]. This suggests the wide range of uses for BPs. In this study, we systematically elaborated the mechanism of the effects of BP-1 and BP-3 on human health based on the BAPG-chain model (Figure S2). The BAPG-chain model evaluates cytotoxicity in terms of biochemical detection, the antioxidant defense system, proteins, and genes.

### 2.1. Cell Viability and Morphology

**Figure 2:**
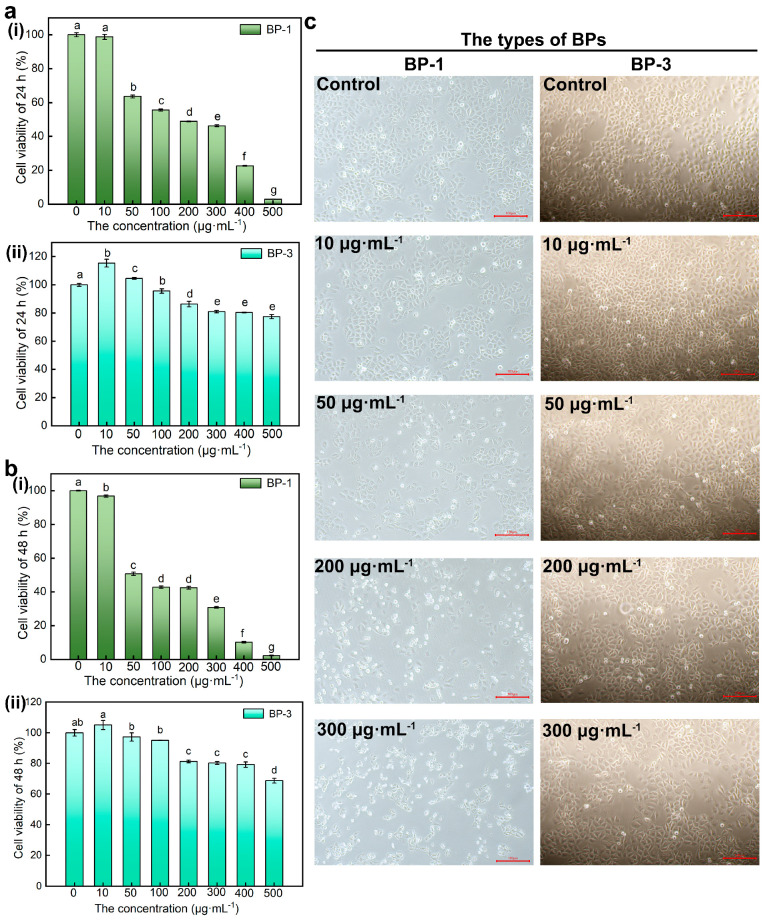
The effect of BPs with different concentrations on cell viability and morphology. (**a**): The effect of BP−1 with different concentrations on cell viability (Lowercase letters a–g denote *p* < 0.05 as obtained using one-way analysis of variance) (**i**) Cell viability of 24 h, (**ii**) Cell viability of 48 h. (**b**): The effect of BP−3 with different concentrations on cell viability (Lowercase letters a–e denote *p* < 0.05) (**i**) Cell viability of 24 h, (**ii**) Cell viability of 48 h. (**c**): The effect of BPs with different concentrations on cell morphology (Scale bar: 100 μm).

Viability assays are vital steps in toxicology that explain the cellular response to a toxicant. Also, they give information on cell death, survival, and metabolic activities. The results showed that the cell viability of SMMC-7721 cells decreased with higher concentrations of BPs and longer intervention times (Figure 2a,b). With the increase in concentration, the inhibitory effect of BP-1 on cell viability increased, while the inhibitory effect of BP-3 on cell viability increased first and then decreased. First, numerous results show that activity duality is dose-dependent [15,16]. This result showed that BP-3 has a growth-promoting effect in very low concentrations (hormesis) [17], which appears only in very low concentrations and over a very narrow range, and exhibits toxicity at higher doses. Besides this, BP-1 causes apoptosis at very low concentrations. It can be seen that BP-1 has a more substantial inhibitory effect on cell viability than BP-3. According to the CCK-8 results, BP concentrations of 0 μg·mL^−1^, 10 μg·mL^−1^, 50 μg·mL^−1^, 200 μg·mL^−1^, and 300 μg·mL^−1^ were selected as the treatment conditions for subsequent experiments to treat the cells for 24 h.

The earliest and most obvious effect of exposing cells to hazardous compounds is alterations in cell shape or morphology. The cells of control groups were complete, well-defined, and mostly shuttle-shaped in morphology (Figure 2c). The cell density decreased, the floating cells increased in number and became smaller, and most of the cells became round and blurred in outline with higher concentrations of BP-1. With higher concentrations of BP-3, the cell density increased at low concentrations with a small number of rounded cells, probably due to natural apoptotic cells during cell growth and reproduction, and decreased at high concentrations with enhanced cell crumpling. The results showed that cell morphology was altered and cell damage occurred after BP-1 and high concentrations of BP-3 treatment.

### 2.2. Cell Apoptosis

To assess the extent and mode of cell death, a Hoechst 33342 staining experiment was carried out. Hoechst 33342 is a fluorescent dye that can pass through cell membranes and can be used to stain cell nuclei. The cells of control groups fluoresced uniformly with large and full nuclei and smooth nuclear membranes with a dark blue distribution (Figure S3a). With higher concentrations of BP-1, the cells were stained bright blue, the nuclei were agglomerated or fragmented, and dense staining appeared at high concentrations, showing typical apoptotic features (Figure S3a). The cells treated with low concentrations of BP-3 showed dark blue staining, while high concentrations enhanced cell staining with a bright blue color (Figure S3a). It can be seen that the nuclear fluorescence intensity of the cells in BP-1 groups increased, while that of BP-3 groups decreased first and then increased (Figure S3b).

The primary sources of ROS in cells include mitochondria, endoplasmic reticulum, and peroxisomes. Meanwhile, various factors, such as cytokines and free fatty acids, can also trigger the production of ROS [18,19]. The production of ROS in cells is in a dynamic balance; a small amount of ROS can promote cell proliferation and differentiation. When the dynamic balance is broken, a large amount of ROS can cause oxidative stress damage to cells, leading to apoptosis [20]. The level of ROS was significantly increased in BP-3 and BP-4-exposed inverted unicellular green algae (*C. reinhardtii*) cells, leading to mitochondrial dysfunction and cell apoptosis [21]. DCFH-DA acts as a fluorescent probe that can sensitively detect intracellular ROS levels. With higher concentrations of BP-1, the intensity of the green fluorescence in the cells gradually increases, and the number of live cells decreases (Figure S3c). It can be seen that ROS levels were elevated in the BP-1 group (Figure S3d). The green fluorescence intensity of cells in the BP-3 group decreased first and then increased (Figure S3c). It can be seen that the ROS level in the BP-3 group decreased first and then increased (Figure S3d). The reason is that the cells were not damaged after BP-3 intervention at low concentrations. The cells grew, multiplied, and increased in number, so the level of intracellular ROS decreased. In contrast, high concentrations of BP-3 resulted in cellular damage. A large number of free radicals were produced in the cells, and the oxidative stress system was disturbed. The cells were unable to eliminate ROS in time, which increased the level of intracellular ROS. These results indicated that the massive production of ROS is one of the causes of apoptosis after BP intervention.

### 2.3. Changes in Mitochondrial Membrane Potential and Ultrastructure

Normally, the mitochondrial inner membrane potential is high and remains at a negative potential, while the outer membrane potential is low and remains at a positive potential. When the cell is subjected to external stimuli, this will lead to mitochondrial respiratory chain electron-transfer process obstacles, affecting the formation of a proton (H^+^) transmembrane gradient in the stroma, which will lead to the outer positive, inner negative mitochondrial membrane potential to decline, greatly affecting the biological activity in the cell. This can lead to apoptosis.

**Figure 3:**
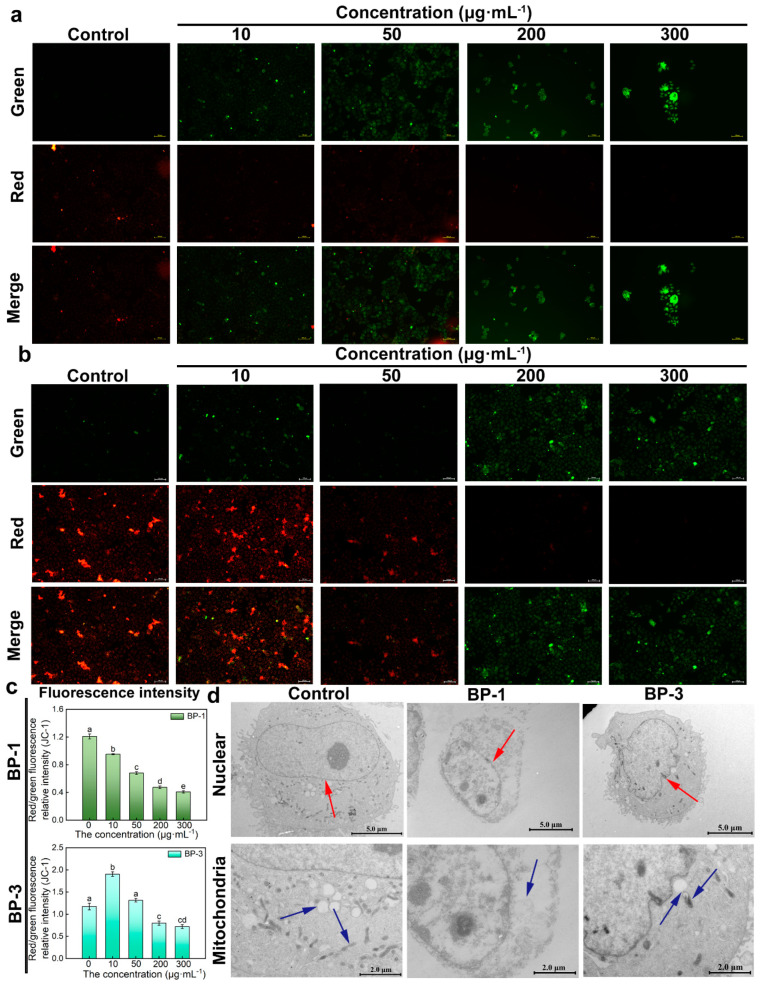
Changes in mitochondrial membrane potential (MMP) and ultrastructure. (**a**): The effect of BP-1 on MMP (Scale bar: 100 μm). (**b**): The effect of BP-3 on MMP (Scale bar: 100 μm). (**c**): Relative intensity of red/green fluorescence of BPs (Lowercase letters denote *p* < 0.05). (**d**): Ultrastructure of cells in the presence of BPs (Scale bar of nuclear: 5.0 μm; Scale bar of mitochondria: 2.0 μm) (The red arrow points to the nucleus, and the blue arrow points to the mitochondria).

JC-1 dye accumulates in mitochondria in a potential-dependent manner. In normal mitochondria, JC-1 accumulates in the mitochondrial matrix to form polymers that emit intense red fluorescence. When an external stimulus induces a collapse of the mitochondrial membrane potential, JC-1 can only exist in the cytosol as a monomer due to a decrease or loss of membrane potential, emitting green fluorescence. Therefore, the ratio of red/green fluorescence intensity can be used to measure the degree of mitochondrial membrane potential change. The results showed that, compared with control groups, the green fluorescence of cells was significantly enhanced, and the red fluorescence was significantly weakened with higher concentrations of BP-1 (Figure 3a,b). The green fluorescence showed “ascending and then descending” patterns with higher concentrations of BP-3. The relative intensity of the red/green fluorescence decreased, and that of the BP-3 groups increased and then decreased with higher concentrations of BPs. The results showed that the mitochondrial electron transfer process was impaired after the intervention with 10 ug·mL^−1^; the membrane potential decreased, the biological activity was reduced, and even some of the cells died by apoptosis. The mitochondrial membrane potential was not affected by the intervention with 10 ug·mL^−1^ BP-3, while the cells showed normal growth and reproduction processes.

The effects of BPs on apoptosis were investigated via the observation of the ultrastructure of the cells exposed to BPs (Figure 3d). The cells of control groups were regular in morphology, oval or round in shape, and large. The nucleus was round-like, and the nuclear membrane was intact. The nucleoli were rounded and clear, and the cytoplasm was rich in mitochondria. The BP groups had irregular cell morphology and reduced cell volume. The nuclear membrane of the cells was ruptured and disappeared, and mitochondrial structures were lost with the highest concentration of BP-1. The nuclei were irregular in shape, with depressions on the surface, and the nucleoli were solidly constricted, but well-defined with the highest concentration of BP-3. The number of mitochondria was reduced, and the intra-membrane matrix was partially dissolved; cristae disappeared and vacuolation occurred.

### 2.4. Level of Antioxidant Defense System

Overall, oxidative stress occurs due to the excessive activity of oxidative enzymes or dysfunction of the antioxidant enzyme system [22]. When the cells are subjected to external environmental stress, many oxygen radicals are produced in the cell, disrupting the dynamic balance of free radical production and elimination. The production of free radicals attacks the intracellular membrane structure and causes enhanced lipid peroxidation, and MDA is one of the essential products of lipid peroxidation in the cell membrane. Meanwhile, SOD, CAT, GSH-Px, and other antioxidant enzyme systems can catalyze the body’s superoxide anion (O_2_^−^) to disproportionate it and maintain the dynamic balance of oxygen radicals [23]. At the same time, they can also reduce specific amounts of fat-soluble and water-soluble compounds [24], preventing the body from increasing the degree of oxidative stress. It was found that BP exposure altered SOD, CAT, and GST activity and glutathione (GSH) levels, leading to oxidative stress in Staphylococcus aureus and fish liver [25]. The activities of SOD, CAT, GSH-Px, and GSH in zebrafish liver treated with BP-3 were significantly decreased [26]. Lactate dehydrogenase (LDH) is present in the cytoplasm under normal conditions. When the structural integrity of the phospholipid bilayer of the cell membrane is disrupted, LDH leaks across the incomplete cell membrane into the culture medium. The extent of cell damage can be reflected by measuring LDH activity in the cell culture supernatant. The antioxidant enzyme activities in the cells all showed a dose–effect relationship after exposure to different concentrations of BPs (Figure S4a). The activity of SOD, CAT, and GSH-Px enzymes were down-regulated, LDH enzyme activity was enhanced, and MDA content was increased with higher concentrations of BP-1. This indicated that BP-1 inhibited the antioxidant capacity of hepatocytes, disrupted the cell membrane structure, and allowed LDH to leak out, ultimately causing oxidative stress in hepatocytes. With higher concentrations of BP-3, the activities of SOD, CAT, and GSH-Px enzymes showed an “inverted U” pattern, and the LDH enzyme activity and MDA content showed an overall increase. This indicated that the low concentration of BP-3 did not cause oxidative stress in the cells, but when the concentration of BP-3 reached a certain level, the cells started to show oxidative stress.

It should be pointed out that ROS was produced primarily via an enzymatic reaction. Enzymes are highly selective for substrates, and it is difficult for the redox reactions of BPs to target the generation of specific ROS in complex cellular environments if it is not based on specific enzymes. Also, intracellular pH, temperature, and ionic strength are usually stabilized in the neutral range, whereas the direct redox reaction of BPs to produce ROS often requires extreme conditions (e.g., strong acids/bases or high temperatures), which is difficult to meet in the physiological environment. The redox of BPs for ROS production may require high concentrations of substrates or cofactors, which is difficult to achieve in cells, whereas enzymatic reactions can be carried out at low substrate concentrations (as described by the kinetic properties of the Mie equation). Cells precisely control ROS levels by regulating enzyme expression, inhibitors, or activators, while most studies have confirmed that ROS originate mainly from enzymatic pathways through enzyme inhibitor or gene knockout experiments of enzymes.

Meanwhile, BPs can bind mainly to SOD1, CAT, GPX1, and LDH-A proteins by van der Waals forces and hydrogen bonds. The toxicological mechanisms of BPs were informed by the critical interactions of BPs with the main enzymes of the antioxidant defense system. BPs bind to the four enzyme proteins at different sites (Figure S4b,c). BP-1 mainly bound to SOD1 protein by van der Waals forces, while Glu 21, Glu 24, Gly 12, Gly 16, Ile 17, and Phe 20 formed a hydrophobic closed region to encapsulate BP-1 (Figure S4b(i)). BP-3 was mainly bound to SOD1 protein by van der Waals forces and hydrogen bonds, BP-3 was wrapped in a hydrophobic pocket formed by the Ile 35, Gly 44, His 48, Glu 40, Gly 51, and Phe 50 of the SOD protein, and BP-3 formed hydrogen bonds with His 48 and was also surrounded with amino acids such as Ile 35 and Glu 40 by van der Waals forces to assist in the binding process (Figure S4c(i)). BPs were mainly bound to CAT protein by van der Waals forces and hydrogen bonds. BP-1 formed hydrogen bonds with Val 73 and Phe 334, and was also surrounded via van der Waals forces by amino acids such as Val 74, Arg 112, Ala 333, and Tyr 358 to assist in the binding process (Figure S4b(ii)). BP-3 formed hydrogen bonds with Arg 72 and was also surrounded via van der Waal forces by amino acids such as Phe 64, Ser 114, Thr 361, and Asp 65 to assist in the binding process (Figure S4c(ii)). In Figure S4c(iii), BP-1 was bound to residue Leu 109 in GPX1 protein by hydrogen bonds, while the surrounding residues Lys 95, Phe 107, Leu 22, Met 108, Ala 23, and Phe 203 formed van der Waals-assisted binding interactions. As shown in Figure S4d(iii), BP-3 was mainly bound to GPX1 protein by van der Waals and hydrophobic forces. BP-3 was wrapped in a hydrophobic pocket formed by Pro 21, Leu 22, Phe 107, Phe 103, Leu 94, Met 108, Leu 109, Pro 105, Leu 91, Glu 104, and Lys 95 in GPX1. BPs mainly bound to LDH-A protein through van der Waals forces and hydrogen bonds. BP-1 formed hydrogen bonds with Ala 29 and Tyr 246, surrounded by amino acid residues such as Ala 33, Trp 249, Met 32, and Glu 60 to form van der Waals-assisted binding interactions, as well as different types of hydrophobic interactions with Trp 249 and Tyr 246 (Figure S4b(iv)). BP-3 was wrapped in a hydrophobic pocket formed by Phe 118, Ile 119, Val 50, Gly 26, Val 25, Asn 114, Gly 96, Ile 115, Asp 51, Ala 95, Tyr 82, and Val 52 in LDH-A. BP-3 formed hydrogen bonds with Tyr 82 and was also surrounded via van der Waals-assisted binding by Phe 118, Gly 96, and Val 25 (Figure S4c(iv)). The results of molecular docking showed that the different docking results of BP-1 and BP-3 with the four protein molecules were mainly due to methoxy. Because methoxy is an electron-donating group, the charge density is relatively large and it enhances the nucleophilicity of negative ions. Therefore, methoxy can make the benzene ring more electrophilic through the electron-withdrawing effect, thus promoting the binding of the benzene ring to the protein. Meanwhile, methoxy is used as the neighboring para-localization group to increase the electron cloud density of the neighboring para-carbon atom more.

The binding energy of BPs interacting with the main enzymatic proteins of the antioxidant defense system is shown in Table S2. The binding energy was less than zero, indicating that the receptors and the ligand can bind spontaneously. The higher the absolute value of the binding energy, the stronger the docking ability and the higher the stability of the molecule after docking. The docking results showed that the binding energy of BPs with the four main enzyme protein receptors was less than −5 kcal·mol^−1^, indicating that BPs could form a stable bond with the enzyme protein. The order of the binding stability of BPs to the main enzyme proteins was: CAT > LDH-A > GPX1 > SOD1.

Molecular docking simulation theoretically illustrated the binding sites of BPs with the main enzyme proteins and the types of forces in the system, including van der Waals forces and hydrogen bonds, assisted by hydrophobic forces. Antioxidant defense system-level assays and molecular docking simulations revealed the causes of BPs’ toxicity to the cells at the surface and microscopic levels.

### 2.5. Gene Expression and Protein Expression

It has been shown that mitochondria are closely related to apoptosis, and that once the MMP collapses, apoptosis is irreversible. Decreases in the MMP have been shown to be one of the earliest responses in the apoptotic cascade, and is known as the “life and death switch” of apoptosis. Apoptosis is a naturally occurring process of abnormal programmed directed cell death, and the mitochondrial pathway has an important role in the apoptotic process. The Bcl-2 family is significant in the mitochondrial pathway [27], and the Bcl-2 family includes the anti-apoptotic gene/protein Bcl-2 and the pro-apoptotic gene/protein Bax. Bax generally exists in the cytoplasm, and Bcl-2 exists in the mitochondrial membrane. When the mitochondrial apoptotic pathway is activated, the activation of Bax and Bcl-2 will lead to changes in mitochondrial membrane permeability. Cytochrome C is released into the cytoplasm and starts the Caspase cascade reaction. The activated Caspase-3 can directly degrade the intracellular structural and functional proteins, making the apoptosis enter the irreversible stage [28,29]. It was found that BP-1 exposure caused mitochondrial damage and reduced red fluorescence in human keratin-forming cells (HaCaT cells), Bcl-2 mRNA was significantly down-regulated, and Bax mRNA was significantly up-regulated [30].

With higher concentrations of BP-1, *Bcl-2* mRNA was gradually down-regulated, and *Bax* mRNA and *Caspase-3* mRNA were gradually up-regulated (Figure S5a). The differences in *Bcl-2* mRNA and *Caspase-3* mRNA were not significant at concentrations of 50 μg·mL^−1^ and 200 μg·mL^−1^. *Bcl-2* mRNA was down-regulated first and then up-regulated. However, *Bax* mRNA and *Caspase-3* mRNA showed an “inverted U” pattern with higher concentrations of BP-3. There were significant differences in *Bcl-2* mRNA and *Caspase-3* mRNA (*p* < 0.05), and the differences in *Bax* mRNA at high concentrations were not significant. The results showed that BPs can cause apoptosis by regulating *Bcl-2* family gene expression and releasing the *Caspase* family.

The expression of the internal reference protein β-actin was similar in the experimental groups, indicating that the total protein content was the same in each group (Figure S5b). Compared with the control groups, Bcl-2 protein expression was decreased. Bax protein expression was increased in the BP-1 group (Figure S5b). The Bcl-2/Bax protein expression ratio was reduced in a dose-dependent manner, indicating that the cells entered the mitochondrial apoptosis process after BP-1 treatment. Compared with the control group, the Bcl-2 protein expression in the BP-3 group first increased and then decreased, the Bax protein expression increased, and the Bcl-2/Bax protein expression ratio decreased (Figure S5b).

### 2.6. RNA Sequencing Analysis

**Figure 4:**
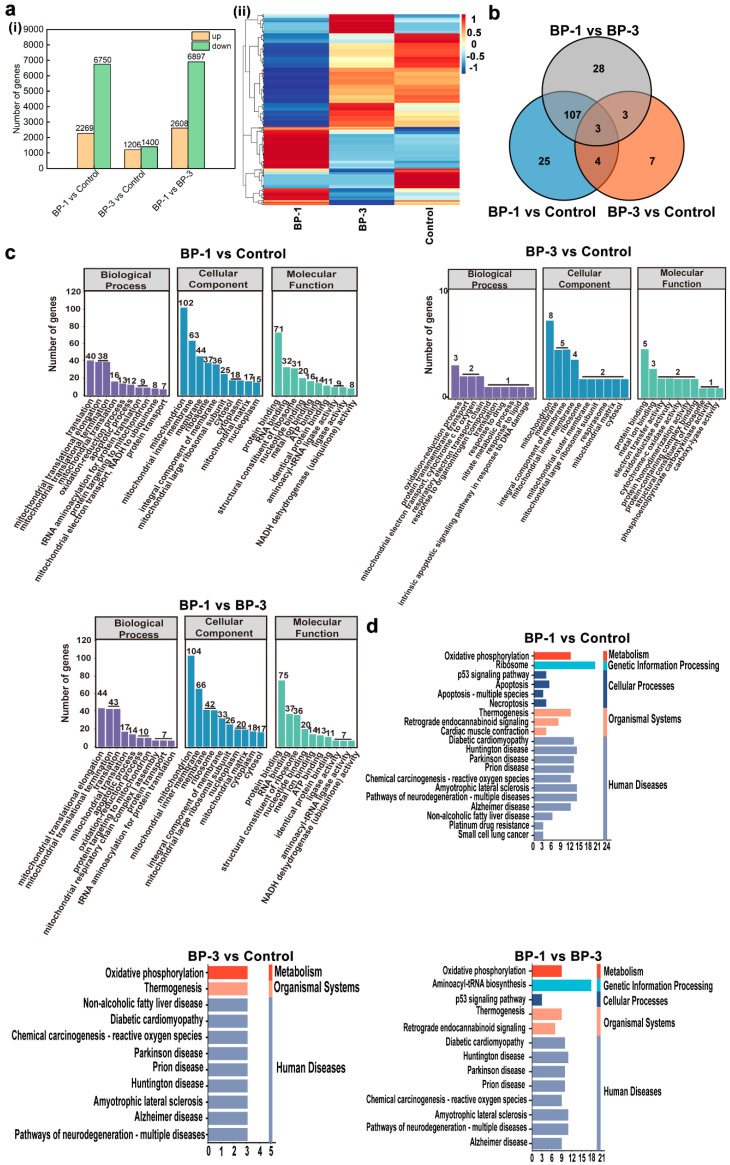
RNA sequencing analysis of SMMC-7721 cells after BP intervention. (**a**): DEGs in different groups. (**b**): Venn diagrams of significantly DEGs related to mitochondria, oxidative stress, and apoptosis. (**c**): GO functional analysis of significantly DEGs. (**d**): KEGG pathway enrichment analysis of significantly DEGs. ((**i**) Number of DEGs in different groups, (**ii**) Heat map of DGEs related to mitochondrial function, oxidative stress, and apoptosis).

Systematic analysis based on transcriptome sequencing and bioinformatics revealed differences in the GO functional enrichment of three related DEGs after the action of BPs. In addition, although the KEGG pathway was enriched in apoptosis-related pathways in the three comparison groups of DEGs, the pathways responsible for apoptosis were different. In this study, the transcriptome was sequenced for each treatment group and differential expressed genes (DEGs) between the different treatment groups were analyzed (Figure 4a). Comparing the BP-1 groups and control groups, there was a total of 9017 DEGs, with 2269 DEGs up-regulated and 6750 DEGs down-regulated. A comparison of the BP-3 groups and control groups revealed a total of 2606 DEGs, with 1206 DEGs up-regulated and 1400 DEGs down-regulated. A total of 8505 DEGs were obtained by comparing the BP experimental groups, with 2608 DEGs being up-regulated and 6897 DEGs being down-regulated.

Based on physiological and biochemical assays, it was shown that BPs could lead to apoptosis. The primary manifestation was a decrease in MMP due to mitochondrial damage, which induces cellular oxidative stress. Furthermore, the gene and protein expression of the Bcl-2 family were altered. Therefore, this study searched for DEGs associated with mitochondria, oxidative stress, and apoptosis in the transcriptome DEGs. The results screened 339 DEGs related to mitochondria, four DEGs related to oxidative stress, and 47 DEGs related to apoptosis (Figure 4a). Three overlapping significant DEGs were in the Venn diagrams’ three comparison groups. This indicates that most genes causing differential changes in the cells by BPs are different, predicting different target molecules or mechanisms of toxic effects.

According to the functions of genes, DEGs are mainly classified into three categories: Biological Process (BP), Cellular Component (CC), and Molecular Function (MF). BP describes biological processes and more closely resembles biological phenotypes. CC describes the localization of genes on cells. MF describes the mode of protein action and focuses more on molecular function. In order to further analyze and compare the distribution functions and the pathways of action of the significant DEGs in the three comparative groups, we performed GO enrichment analysis on the significant DEGs. The biological functions and metabolic pathways involved in the enrichment of all the significant DEGs in the three comparison groups were analyzed to elucidate the relevant analytical mechanisms for the role of BP exposure. The top 10 GO terms enriched by the three comparison groups are shown in Figure 4 (*p* < 0.05). GO analysis showed that in the BP category, the functions of the significant DEGs in the three comparison groups were mainly focused on biological processes such as the Oxidation–reduction process and Translation. In the CC category, the functions of the significant DEGs in the three comparison groups were mainly concentrated in the Mitochondrion, Membrane, Integral component of membrane, Mitochondrial inner membrane, Ribosome, Mitochondrial matrix, and Cytosol. In the MF analysis, the functions of the three comparison groups of significant DEGs were mainly focused on Protein binding, Metal ion binding, Structural constituent of the ribosome, and other functions. The GO enrichment results of the three comparison groups with significant DEGs indicated that BPs mainly induced a series of physiological and biochemical changes in the process of apoptosis, involving a variety of substance synthesis and binding processes.

The enrichment of the KEGG pathway for the three comparison groups with significant DEGs (*p* < 0.05) is shown in Figure 4. The results of the KEGG pathway enrichment showed that the KEGG enriched in the three comparison groups contained different metabolic pathways and the same metabolic pathway. The different metabolic pathways mainly included Ribosome, Aminoacyl-tRNA biosynthesis, Apoptosis, Apoptosis-multiple species, Necroptosis, Cardiac muscle contraction, Non-alcoholic fatty liver disease, Platinum drug resistance, small cell lung cancer, and other pathways. The same metabolic pathways mainly included Oxidative phosphorylation, the *p53* signaling pathway, Chemical carcinogenesis-reactive oxygen species, Thermogenesis, Diabetic cardiomyopathy, Huntington’s disease, Parkinson’s disease, and so on. It is thus clear that both BPs can lead to apoptosis by inducing the cell cycle as well as by reducing the content of active substances. In addition, as BPs lead to significant targeting, DEGs also exist in different metabolic pathways, suggesting that there were also different signaling pathways for BPs to lead to apoptosis.

There were 38 up-regulated DEGs and 121 down-regulated DEGs in the three comparison groups (Figure S6a). The 159 DEGs obtained by filtering were imported into the String website to obtain the PPI (Figure S6b), and a total of 131 nodes and 1014 edges were obtained. There were 21 Hub genes in the PPI obtained by screening with degree, betweenness, and closeness averages as thresholds (Figure S6c).

These findings led to a better understanding at the molecular level of the different cytotoxic effects induced by BPs on the cells and the different molecular mechanisms and pathways of action involved.

Meanwhile, the Bcl-2 family is significant in the mitochondrial pathway [31], and the Bcl-2 family includes the anti-apoptotic gene/protein Bcl-2 and the pro-apoptotic gene/protein Bax. Bax generally exists in the cytoplasm, and Bcl-2 exists in the mitochondrial membrane. When the mitochondrial apoptotic pathway is activated, the activation of Bax and Bcl-2 will lead to changes in mitochondrial membrane permeability. Cytochrome C is released into the cytoplasm and starts the Caspase cascade reaction. The activated Caspase-3 can directly degrade the intracellular structural and functional proteins, making the apoptosis enter the irreversible stage [30,31]. Our results indicated that the exposure of BPs to SMMC-7721 cells activated the Bax/Bcl-2/Caspase-3 signaling pathway and directly induced apoptosis.

A recent study showed that BP-3 induces apoptosis through RXR-mediated signaling pathways, thereby affecting zebrafish locomotion and response capabilities [32]. Another study showed that BP-3 affects the nervous system by regulating calcium signaling pathways [11]. In addition, BP-3-treated zebrafish show significant differences in AChE gene expression and its activity [33]. Therefore, BP-1 and BP-3 can not only stimulate cellular oxidative stress leading to apoptosis, but can also lead to apoptosis by pathways such as endocrine system disruption.

## 3. Materials and Methods

### 3.1. Materials and Equipment

BP-1 and BP-3 (>99%) were purchased from Tokyo Chemical Industry (Tokyo, Japan). Human hepatoma SMMC-7721 cells were purchased from the Library of Tumor Cells of the Chinese Academy of Medical Sciences (Beijing, China). Hepatocellular carcinoma cell lines, such as SMMC-7721 derived from human tissues, serve as optimal in vitro models for studying BP-1 and BP-3 toxicity mechanisms. Their rapid proliferation and vigorous growth, combined with shared oxidative stress and apoptosis pathways with normal hepatocytes, enable the effective assessment of human cellular responses to these compounds. The Supporting Information (SI) contains further information about the materials and equipment required for the experiments, solution preparation, and cell cultures.

### 3.2. CCK-8 Assay

The cells were exposed to BP-1 and BP-3 working solutions for 24 h and 48 h. CCK-8 reagent was added and the plates were incubated for 0.5 h. Cell viability was calculated by measuring the absorbance at 450 nm. All specific details concerning the CCK-8 assay can be found in the SI.

### 3.3. Cytomorphology Analysis

After exposure of the cells to appropriate concentrations of BP-1 and BP-3 for an appropriate time according to the CCK-8 assay, cell morphology was observed by an inverted microscope (100×).

### 3.4. Apoptosis Assay

According to the manufacturer’s instructions, the cells of different groups were stained with the Hoechst 33342 staining solution, observed under an inverted fluorescent microscope.

### 3.5. ROS Assay

According to the instructions of the ROS assay kit, the H_2_DCFDA working solution (10 μmol·L^−1^) was used for the staining. After the cells were washed twice with phosphate buffered saline (PBS), 1 mL PBS was added to each well and observed by fluorescence microscope.

### 3.6. MMP Assay

According to manufacturer’s instructions, the JC-1 working solution was added to each well of 12-well plates, then the plates were incubated in the incubator for 20 min in the dark. The plates were observed under an inverted fluorescent microscope.

### 3.7. Electron Microscopy

The cells were exposed to BPs and collected in the microcentrifuge tubes. Moreover, the cells were fixed in 2.5% glutaraldehyde overnight, dehydrated, dried, and then embedded in paraffin wax. The slices were stained and dried, and the cellular ultrastructure was observed by transmission electron microscopy (TEM) and photographed.

### 3.8. Measuring the Activity of Antioxidant Enzymes and the Content of the Active Substance

After the cells were exposed to BP-1 and BP-3, the culture was collected in Eppendorf tubes and centrifuged to collect the supernatant, used to determine LDH enzyme activity. The cell lysate-added RIPA lysis buffer was collected by refrigerated centrifugation, used to determine the enzyme activities of catalase (CAT), superoxide dismutase (SOD), and glutathione peroxidase (GSH-Px), as well as malondialdehyde (MDA) content. All specific details concerning measuring the activity of SOD, CAT, GSH-Px, and lactate dehydrogenase (LDH), and measuring the content of MDA, can be found in the SI.

### 3.9. Molecular Docking

As ligands, BP-1 and BP-3 were optimized for MM2 energy with the help of Chem Draw software (version 23.1.1) [34]. The crystal structures of SOD1, GPX1, CAT, and LDH-A were used as receptors. The receptors are opened in Discovery Studio software (version 4.5) for dehydration and hydrogenation pre-processing [35]. The Auto Dock software (version 4.2.6) adds AD4 atom types and saves the files [36]. Molecular docking was performed using Auto Dock software, setting the receptors to rigid for semi-flexible docking. The Lamarckian Genetic Algorithm (LGA) was chosen for the internal conformational search. The binding activity of BP-1 and BP-3 to the stress enzymes was assessed by binding energy. The 2D schematic of the protein–ligand complex was generated using Discovery Studio software. Other specific details concerning molecular docking can be found in the SI.

### 3.10. Evaluation of Gene Expression

The total RNA was extracted from cells using the AxyPrep total RNA miniprep kit, according to the manufacturer’s instructions. The concentration of RNA samples was analyzed by a microRNA analyzer, and the purity and integrity were analyzed by agarose gel electrophoresis. cDNA was obtained by reverse transcription of RNA, and then amplified by PCR. All the primers used in this study are shown in Table S1. The 2^−∆∆Ct^ method was used to calculate the relative expression of *Bax* and *Bcl-2*, using *β-actin* as the internal reference.

### 3.11. Evaluation of Protein Expression

After the cells were treated, the lysis buffer was extracted and diluted with the aim of obtaining the cell lysate. The lysate was subjected to 12% SDS-polyacrylamide gel electrophoresis (SDS-PAGE). Electrophoresis was followed by membrane transfer. Incubation with different antibodies was followed by visualization with ChemiDoc^TM^ XRS.

### 3.12. RNA Sequencing Assay

The cells were exposed to 0.1% DMSO and a concentration of 300 μg·mL^−1^ BP-1 and BP-3 for 24 h. According to manufacturer’s instructions, the total RNA was extracted using the Trizol reagent. Sequencing libraries were constructed using high-quality RNA samples with RIN values > 7.0, and 2 × 150 bp paired-end sequencing (PE150) was performed using Illumina Novaseq™ 6000-raw data with adapter sequences deleted for genomic alignment. Valid data obtained after unqualified, low-quality data and duplicate sequences were removed using Cutadapt (https://cutadapt.readthedocs.io/en/stable/, accessed on 3 January 2025, version: cutadapt-1.9) [37]. Validated data after pre-processing were compared to the human genome library (ftp://ftp.ensembl.org/pub/release-101/, accessed on 3 January 2025) using HISAT2 software (https://daehwankimlab.github.io/hisat2/, accessed on 3 January 2025, version: hisat2-2.2.1) [38] for comparison.

Primary assembly of genes or transcripts was performed using String Tie software [39] and the Ballgown website (http://www.bioconductor.org/packages/release/bioc/html/ballgown.html, accessed on 3 January 2025) [40]. Primary assembly results from all samples were combined. mRNA expression abundance was performed by calculating fragment per kilobase transcript per million mapped reads (FPKM) values. Differential gene expression analysis was performed between the two groups by DESeq2 software [41]. The threshold for DEGs was |log2(foldchange)| ≥ 1, and the false discovery rate (FDR) < 0.05. The screened significantly DEGs were subjected to GO functional classification and KEGG enrichment analysis.

The screened DEGs were imported into the String website (https://www.string-db.org) to construct a protein–protein interaction (PPI) network. The PPI network was imported into Cytoscape software (version 3.10.3) for visualization, and DEGs were analyzed to screen out Hub genes with high protein interaction scores.

### 3.13. Statistical Analysis

All experiments were performed with three replicates, and the results were expressed as mean ± standard deviation (Mean ± Sd). Comparisons between groups were made using a one-way analysis of variance (one-way ANOVA). *p* < 0.05 indicated statistical significance.

## 4. Conclusions

This study presented the BAPG-chain model for the first time. The results showed that BPs caused oxidative stress by stimulating the cells to produce large amounts of ROS. BP-1 and BP-3 interventions induce the production of excess ROS, mainly superoxide anions (O_2_^−^), which disrupts the dynamic balance of enzyme activities in the antioxidant defense system, activating mitochondrial dysfunction, and ultimately leading to apoptosis. This result is consistent with previous evidence that overproduction of superoxide disrupts the mitochondrial pathway of Bax, Bcl-2, and Caspase-3. After intervention with 10 μg/mL BP-1, cell damage occurred. As the concentration increased, the degree of cell apoptosis became more severe. In contrast, when cells were intervened with BP-3 at concentrations below 50 μg/mL, no effect was observed. However, when the concentration exceeded 200 μg/mL, oxidative stress responses occurred in the cells, leading to apoptosis. BPs, as a widely used material in production and life, can enter into organisms through various pathways, thus causing different degrees and mechanisms of damage to different organisms, which requires further attention.

## Data Availability

The additional data supporting the manuscript are available from the corresponding author upon request.

## Supplementary Materials

The following supporting information can be downloaded at: https://www.mdpi.com/article/10.3390/ijms26072990/s1. References [42,43,44,45,46] are cited in the supplementary files.
