# Supplementary material for: Understanding of Benzophenone UV Absorber-Induced Damage and Apoptosis in Human Hepatoma Cells
Source: Int J Mol Sci. 2025 Mar 25;26(7):2990. doi: 10.3390/ijms26072990 (PMC11988835; doi:10.3390/ijms26072990)
Supplement: Supplementary file 1 [file ijms-26-02990-s001.zip › ijms-3450155-supplementary.docx]

**Supporting Information**

**Understanding of Benzophenone UV Absorbers -Induced Damage and Apoptosis in Human Hepatoma Cells**

Luwei Tian^1^, Yanan Wu^1^, Yankun Jia^1,^* , Ming Guo^1,^*

^1^ College of Chemistry and Materials Engineering, Zhejiang Agriculture & Forestry University, Hangzhou, Zhejiang 311300, China

*** Corresponding authors:**

E-mail: guoming@zafu.edu.cn (Ming Guo); jiayk@zafu.edu.cn (Yankun Jia)

**2. Materials and Methods**

*2.1. Materials and equipment*

*2.1.1. Materials*

BP-1 and BP-3 can be enriched in organs such as the liver, kidneys, and spleen through pathways such as skin penetration and environmental exposure [2]. The enrichment leads to the concentration of BPs in the human body that is hundreds of times higher than in the external environment [3]. The liver is an important detoxification organ in the human body that plays a crucial role in the elimination and metabolism of external pollutants [14].

Dimethyl sulfoxide (DMSO) was purchased from Sigma Technology Inc. (Indiana, USA). Hoechst 33342 staining solution for live cells (100×) was purchased from Beyotime Biotechnology (Shanghai, China). Cell counting kit-8 (CCK-8), reactive oxygen species (ROS) assay kit, mitochondrial membrane potential (MMP) detection kit (JC-1), Fluorescent quantitative PCR kit (SYBR green master mix), BCA protein concentration assay kit, and total superoxide dismutase (SOD) assay kit with nitro-tetrazolium blue chloride (NBT) were purchased from Biosharp Co. (Beijing, China). Glutathione peroxidase (GSH-Px) assay kit, malondialdehyde (MDA) assay kit, lactate dehydrogenase (LDH) assay kit and catalase (CAT) assay kit were purchased from Leagene Biotechnology Co., Ltd. (Beijing, China). AxyPrep total RNA miniprep kit was purchased from Axygen (New York, USA). PrimeScript™ RT reagent Kit (Perfect Real Time) was purchased from Takara (Kyoto, Japan). Mouse anti-Bcl-2 monoclonal antibody and mouse anti-β-actin monoclonal antibody were purchased from Applygen Technologies Inc. (Beijing, China). Rabbit anti-Bax monoclonal antibody was purchased from Proteintech Group Inc (Chicago, USA). HRP-labelled goat anti-mouse lgG and HRP-labeled goat anti-rabbit lgG were purchased from Sangon Biotech Co., Ltd (Shanghai, China). Modified RMPI-1640 medium, fetal bovine serum (FBS), phosphate-buffered saline (PBS) (1×), penicillin-streptomycin solution, 0.25% trypsin solution (1×) were purchased from Cytiva (Logan, UT, USA). Other reagents are analytical grade.

*2.1.2. Equipment*

The following equipment was used in this study: HCB-1300V vertical laminar clean table (Haier, Qingdao, China), Thermo DH-800 carbon dioxide culture box (Thermo Fisher Scientific, Massachusetts, USA), CKX31 inverted biological microscope (Olympus, Kyoto, Japan), TI-S fluorescent inverted microscope (Nikon, Tokyo, Japan), iMark 18067 microplate reader (Bio-Rad, Hercules, CA, USA), high-speed refrigerated centrifuge (Eppendorf, Hamburg, Germany), ChemiDoc™ XRS System (Bio-Rad, Hercules, CA, USA), CFX96™ Touch Real-Time PCR Detection System (Bio-Rad, Hercules, CA, USA), electrophoresis instrument and electrophoresis tank (Bio-Rad, Hercules, CA, USA), micropipette (Eppendorf, Hamburg, Germany)

*2.1.3. Solution preparation*

Complete medium was prepared from 10% FBS, 100 international unit·mL^-1^ (IU·mL^-1^), penicillin 100 μg·mL^-1^ streptomycin, and RIPM-1640 medium. 500 mg·mL^-1^ stock solutions of BP-1 and BP-3 were prepared in DMSO, filtered through the 0.22 μm microporous filter to remove bacteria, and stored at -20°C without light. Firstly, the reason is that pollutants from nature reach the human body (the top of the biological chain) at concentrations much higher than those found in nature through bioaccumulation effects. A number of studies have confirmed that it is these bioaccumulation concentrations that are harmful to cells [42,43]. Therefore, the reason for choosing a higher concentration of BP-1 and BP-3 working solutions in the experiment is that BP-1 and BP-3 will enter the human body through a variety of pathways and will be enriched in tissues and organs despite the human blood circulation, resulting in a concentration of BP-1 and BP-3 in tissues that is hundreds of times higher than that in the external environment. Secondly, due to the medium hydrophobicity of BP-1 and BP-3, the concentration of the solution may decay due to surface adsorption when the concentration of the working solution is below 1 mg·L^-1^. It is difficult to make a solution if the concentration is too low. Thirdly, considering the dissociation properties of phenolics (pKa≈8.2), the dissociation constants have a negligible effect on the partitioning behavior (ΔlogKow<0.3) in the physiological pH range (7.0-7.4) where their molecular forms account for >90%. Finally, due to the simplicity and reproducibility of *in vitro* cellular experiments, BP-1 and BP-3 may be degraded in organisms when animal experiments are used, and the relationship between BP-1 and BP-3 and tissues and organs cannot be directly accessed, so *in vitro* cellular methods are used.

Some studies have reported that their concentrations in the urine and blood are much higher than the environmental concentrations [44], which may cause chronic damage to the organism health [45]. It is worth noting that BPs have also been detected in human breast milk and placenta [46]. Hence, the working solutions of BP-1 and BP-3 at concentrations of 10, 50, 100, 200, 300, 400, and 500 μg·mL^-1^ were set. The stock solutions were diluted from complete medium (V_DMSO_: V_Complete medium_ = 0.1%) and stored at 4°C protected from light.

*2.1.4. Cell culture*

SMMC-7721 cells were cultured in a complete medium. The cells were incubated in a 37°C, 5% (V/V) CO_2_ incubator. The cells were washed with PBS, digested with 0.25% trypsin-EDTA digest and passaged at 70-80% confluence.

*2.2. CCK-8 assay*

After resuspending the cultured SMMC-7721 cells, the suspension was counted using a cell counting plate. Cells were seeded at a density of 5.0 × 10^4^ cells⋅mL^−1^ in the 96-well plate and cultured overnight. Blank control groups, experimental control groups, and experimental groups were set up. Cell-free complete medium containing only 0.1% DMSO was used as blank control groups. Cells of experimental control groups were cultured in a complete medium containing 0.1% DMSO. All treatments were performed in triplicate. Cells were cultured for 24 h and 48 h. CCK-8 was then added to the wells, and the plates were incubated for 0.5 h. Cell viability was calculated according to equation (S1) by measuring the absorbance at 450 nm using the microplate reader.

$\text{Cell viability=}\frac{\text{OD}_{\text{Experimental group}}\text{-}\text{OD}_{\text{Blank control group}}}{\text{OD}_{\text{Experimental control group}}\text{-}\text{OD}_{\text{Blank control group}}}\text{×100\%}$ (S1)

Where OD values are the absorbance values of the different groups.

*2.8. Measuring the activity of antioxidant enzymes and the content of active substance*

*2.8.1. Measuring the activity of SOD*

The NBT color reaction was used to measure SOD enzyme activity. The cell lysate was added to the experimental groups. Control group 1 contained SOD buffer and reaction starter solution. SOD buffer was only added to the control groups 2. The plates were incubated at 37°C for 30 min. The absorbance values were measured at 562 nm, and the SOD enzyme activity was calculated according to equation (S2).

$\text{SOD activity }\left( \text{U} \right)\text{=}\frac{\frac{\text{OD}_{\text{Experimental group}}\text{-}\text{OD}_{\text{Control group 1}}}{\text{OD}_{\text{Control group 1}}\text{-}\text{OD}_{\text{Control group 2}}}\text{×100\%}}{\text{1-(}\frac{\text{OD}_{\text{Experimental group}}\text{-}\text{OD}_{\text{Control group 1}}}{\text{OD}_{\text{Control group 1}}\text{-}\text{OD}_{\text{Control group 1}}}\text{×100\%)}}$ (S2)

*2.8.2. Measuring the activity of CAT*

CAT enzyme activity was measured using H_2_O_2_ (65 mmol·L^-1^) as a substrate. The blank groups contained CAT buffer; the cell lysate was added to the experimental control groups after the water bath of 37°C, while the experimental groups were added to the cell lysate before the water bath. One unit of CAT enzyme activity was defined as 1 μmol H_2_O_2_ catalyzed in 1 min at the temperature of 37°C. The CAT enzyme activity was calculated according to equation (S3).

$\text{CAT activity }\left( \text{U·}\text{mg}^{\text{-1}} \right)\text{=}\frac{\left( \text{OD}_{\text{Experimental control group}}\text{-}\text{OD}_{\text{Experimental group}} \right)\text{×650}}{\text{OD}_{\text{Blank group}}\text{×The protein content of cell lysate (mg·}\text{mL}^{\text{-1}}\text{)}}$ (S3)

*2.8.3. Measuring the activity of GSH-Px*

GSH-Px enzyme activity was measured using H_2_O_2_ as a substrate. GSH working solution, cell lysate and oxidation working solution were added to the experimental groups, while the experimental control groups contained no cell lysate. Double-distilled water (ddH_2_O) and acidic precipitant were added to the blank groups. The supernatant was collected by centrifugation at 3500 rpm for 10 min. The groups were added GSH-Px buffer and benzoic acid color solution sequentially and placed at room temperature for 1 min. The absorbance values were measured at 422 nm, and the GSH-Px enzyme activity was calculated according to equation (S4).

$$\text{GSH-Px activity }\left( \text{mU·}\text{mg}^{\text{-1}} \right)\text{=}$$

$\frac{\left( \text{OD}_{\text{Experimental control group}}\text{-}\text{OD}_{\text{Experimental group}} \right)\text{×200}}{\left( \text{OD}_{\text{Experimental control group}}\text{-}\text{OD}_{\text{Blank group}} \right)\text{×The protein content of cell lysate (mg·}\text{mL}^{\text{-1}}\text{)}}$ (S4)

*2.8.4. Measuring the activity of LDH*

The LDH enzyme activity was determined based on the colour reaction of pyruvate with dinitrophenyl hydrazine. The blank groups contained distilled water, and the standards groups contained different concentrations of pyruvate standards. Distilled water and cell lysate were added to the experimental control groups, and the experimental group contained cell lysate and nicotinamide adenine dinucleotide buffer. After the plates were placed at room temperature for 5 min, the absorbance values were measured at 450 nm. The LDH standard curve was constructed using the pyruvate standard as the horizontal coordinate and the OD Standard group-OD Blank group as the vertical coordinates. The LDH enzyme activity was calculated from the standard curve's OD _Experimental group_-OD _Experimental control group_.

*2.8.5. Measuring the content of MDA*

The MDA content was determined by the reaction based on thiobarbituric acid (TBA). In brief, RIPA lysate was used as the blank groups, different concentrations of MDA standards as the standards groups and experimental groups for cell lysate. The samples of the different groups and the MDA assay working solution (containing TBA) were mixed and heated at 95°C for 40 min. The absorbance values were measured at 535 nm after cooling and centrifugation at 4000 rpm for 10 min. The MDA standard curve was constructed using the MDA standard as the horizontal coordinate and the OD _Standard group_-OD _Blank group_ as the vertical coordinate. The MDA content was calculated from the OD _Experimental group_-OD _Blank group_ in the standard curve.

*2.9. Molecular docking*

The SDF format files of BP-1 and BP-3 compounds were downloaded from the Pub Chem website (https://pubchem.ncbi.nlm.nih.gov) and optimized for MM2 energy with the help of Chem Draw software. The PDB Protein Database (http://www.rcsb.org/pdb) exports the SOD1 (PDB ID: 2MP3), GPX1 (PDB ID: 2F8A, resolution: 1.50 Å), CAT (PDB ID: 1DGF, resolution: 1.50 Å), LDH-A (PDB ID: 4L4R, resolution: 2.10 Å) crystal structures were used as receptors. The receptors are opened in Discovery Studio software for dehydration and hydrogenation pre-processing. The Auto Dock software (version 4.2.6) adds AD4 atom types and saves the files. Molecular docking was performed using Auto Dock software, setting the receptors to rigid for semi-flexible docking. The center of the lattice is moved based on the size of the protein binding site until the lattice surrounds the entire active site as a whole, with a default lattice spacing of 0.375 Å. After setting the ambient pH to 7.4, the lattice was set to X:110, Y:110 and Z:110 and the lattice was set to period. The Lamarckian Genetic Algorithm (LGA) was chosen for the internal conformational search. The binding activity of BP-1 and BP-3 to the stress enzymes was assessed by binding energy. The 2D schematic of the protein-ligand complex was generated using Discovery Studio software.

Figure S1


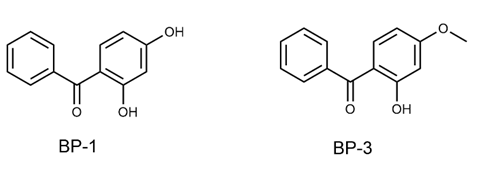


**Figure S1.** Structural formula of BP-1 and BP-3.

Figure S2


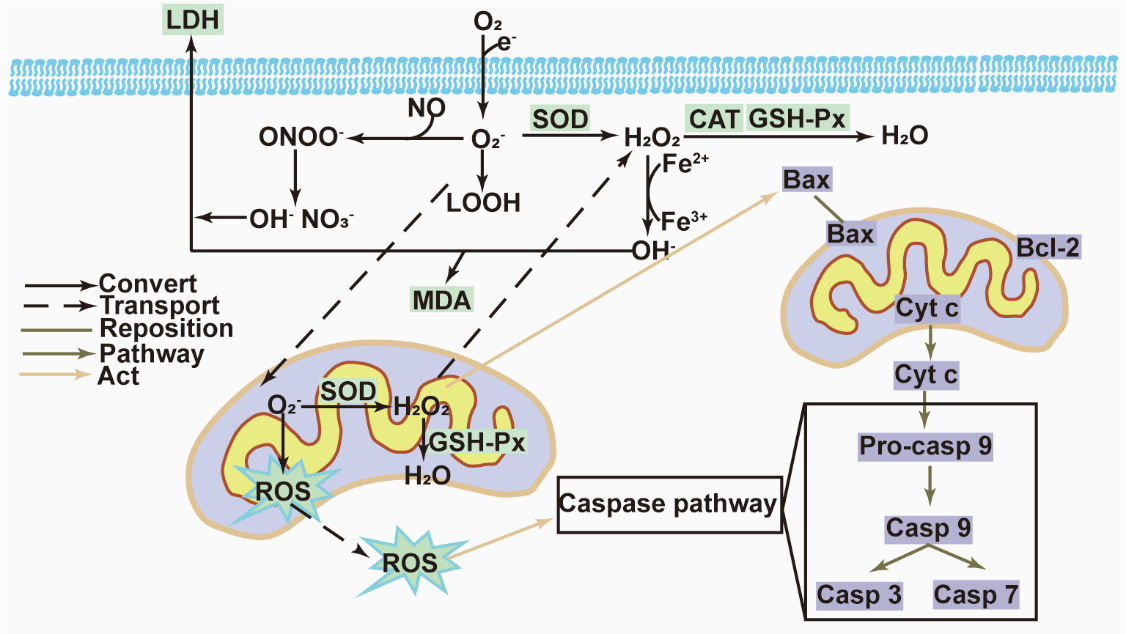


**Figure S2.** Pathway of adverse effects of BPs on the cells.

Figure S3


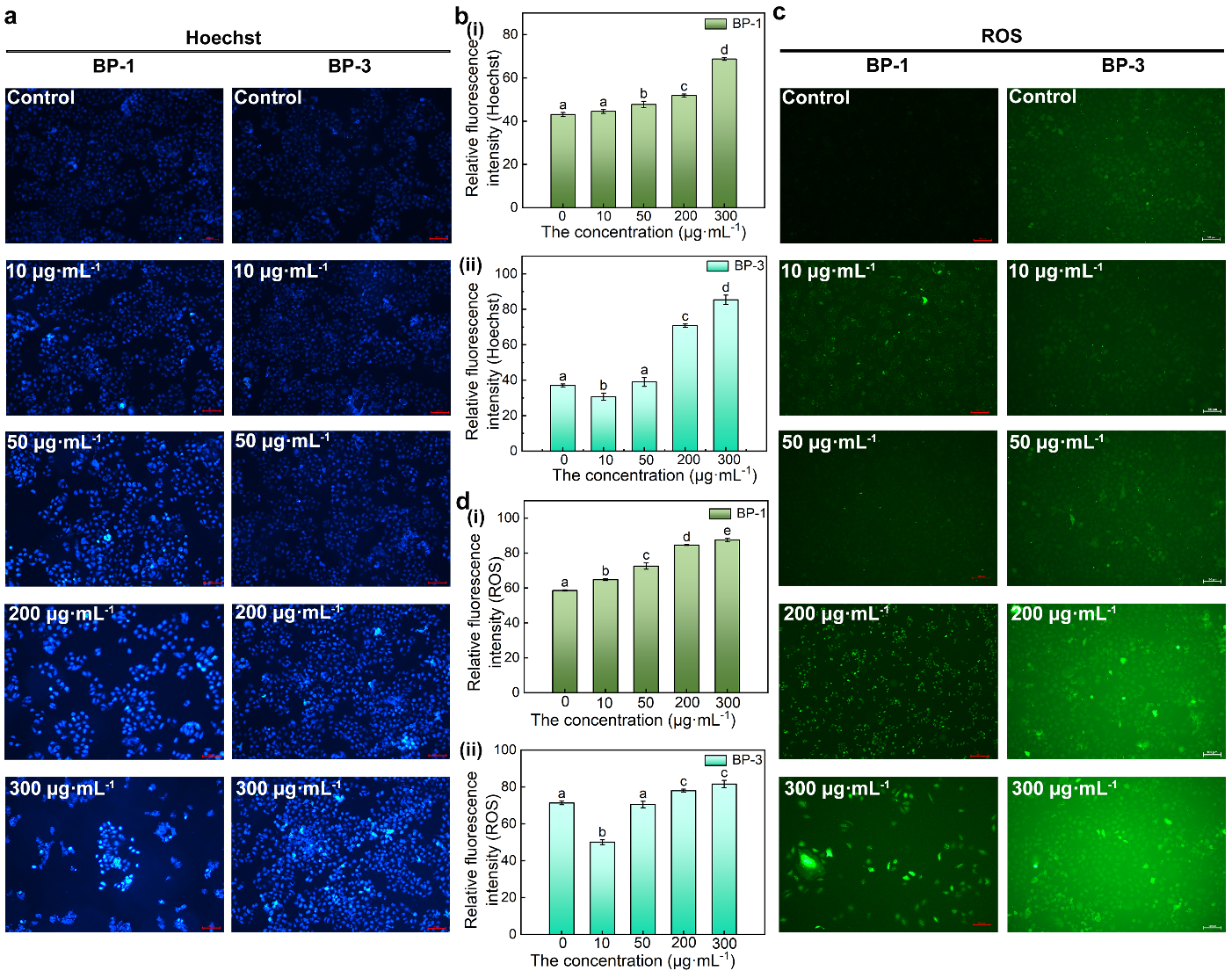


**Figure S3.** The effect of BPs on apoptosis. a: The images results of Hoechst 33342 staining experiment (Scale bar: 100 μm). b: Relative fluorescence intensity of Hoechst 33342 (Lowercase letters denote *p*<0.05) (i) Relative fluorescence intensity in the existence of BP-1, (ii) Relative fluorescence intensity in the existence of BP-3. c: The images results of ROS assay (Scale bar: 100 μm). d: Relative fluorescence intensity detected after ROS assay (Lowercase letters denote *p*<0.05).

Figure S4

**
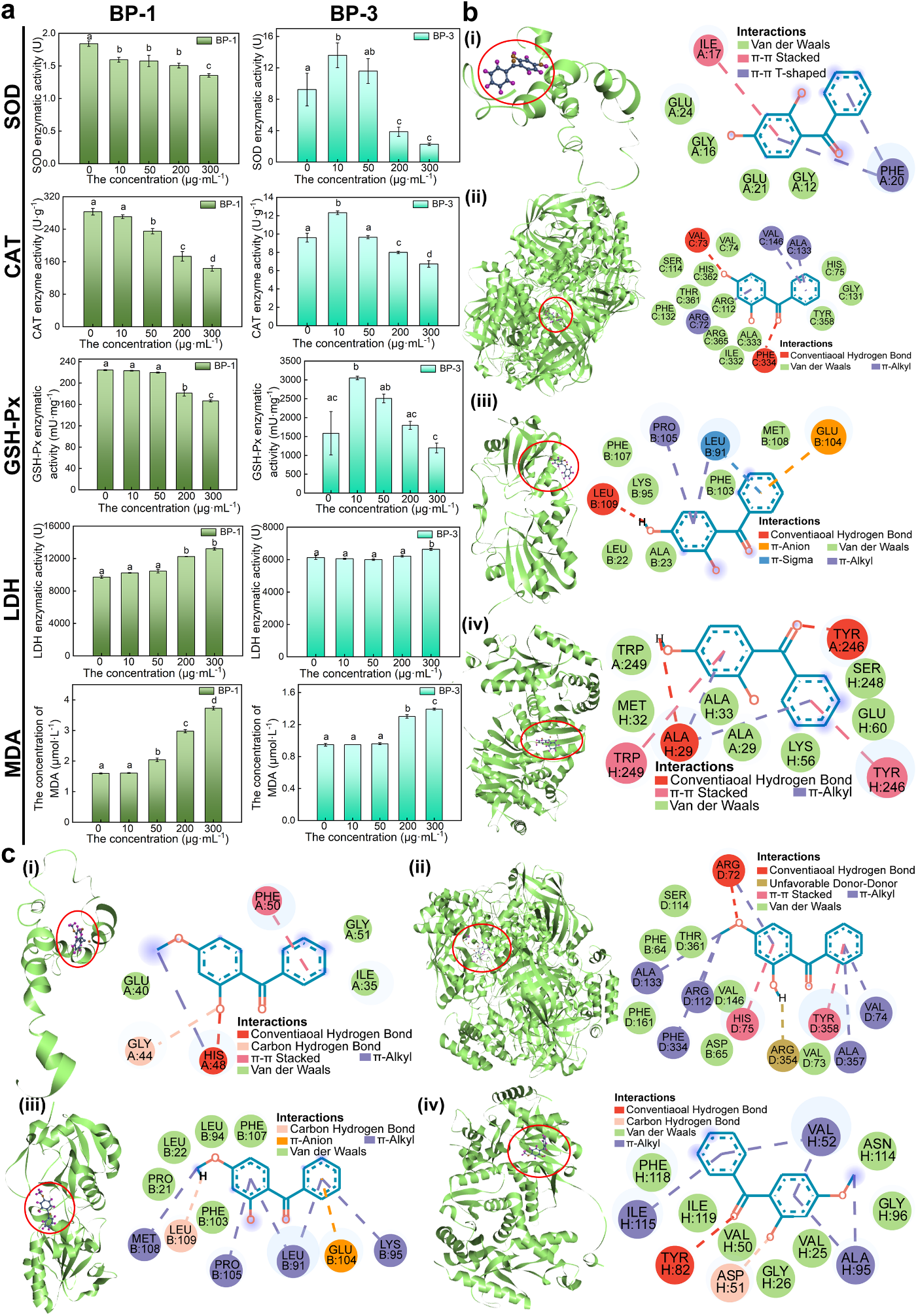
**

**Figure S4.** The effect of BPs on the level of antioxidant defense system. a: The effect of BPs on antioxidant defense system (Lowercase letters denote *p*<0.05). b: Molecular docking diagram of BP-1. c: Molecular docking diagram of BP-3. ((i) SOD1 protein, (ii) CAT protein, (iii) GPX1 protein, (iv) LDH-A protein)

Figure S5


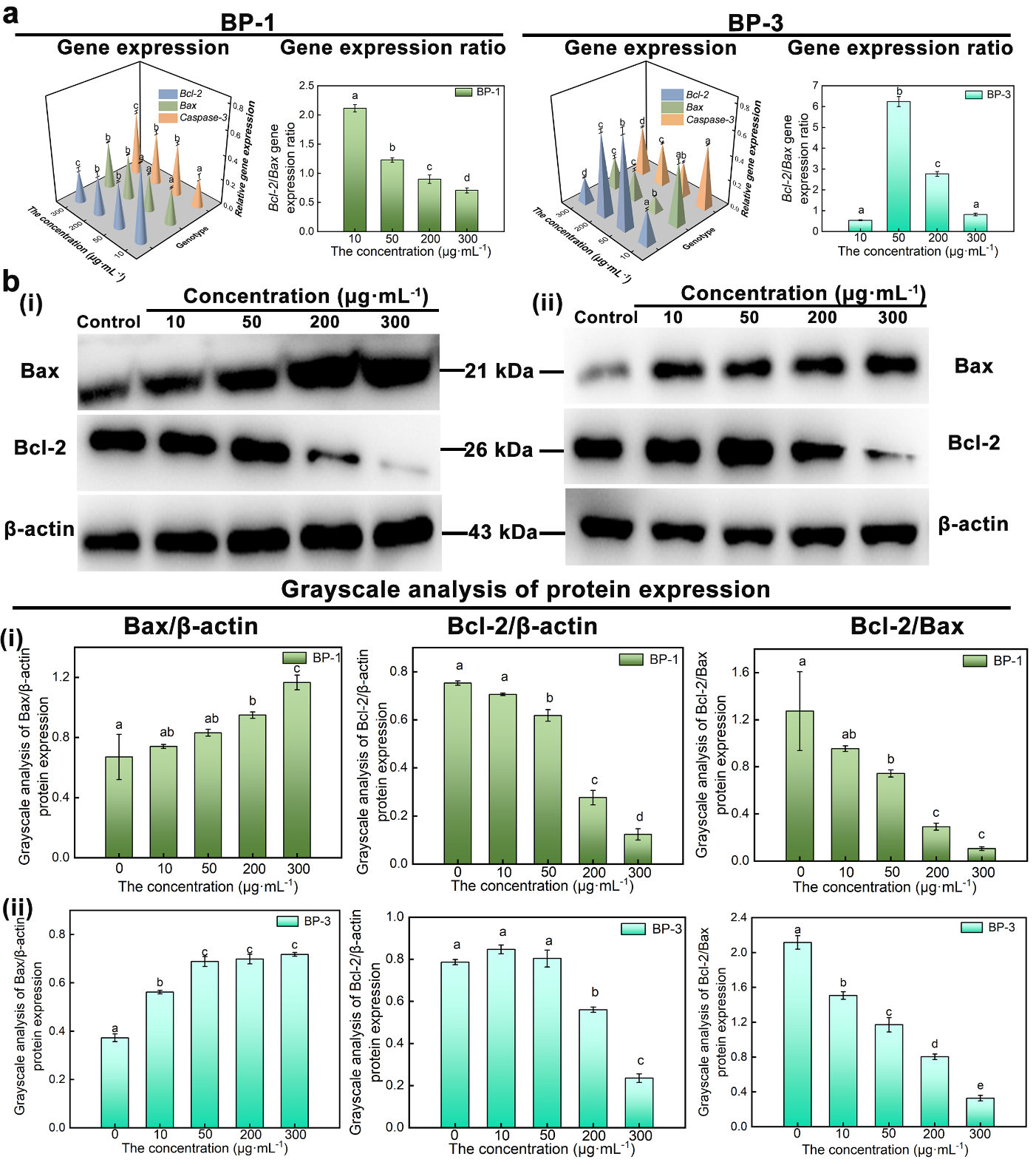


**Figure S5.** The effect of BPs on genes expression and proteins expression. a: The effect of BPs on genes expression (Lowercase letters denote *p*<0.05). b: The effect of BPs on proteins expression (Lowercase letters denote *p*<0.05). ((i) BP-1, (ii) BP-3)

Figure S6

**
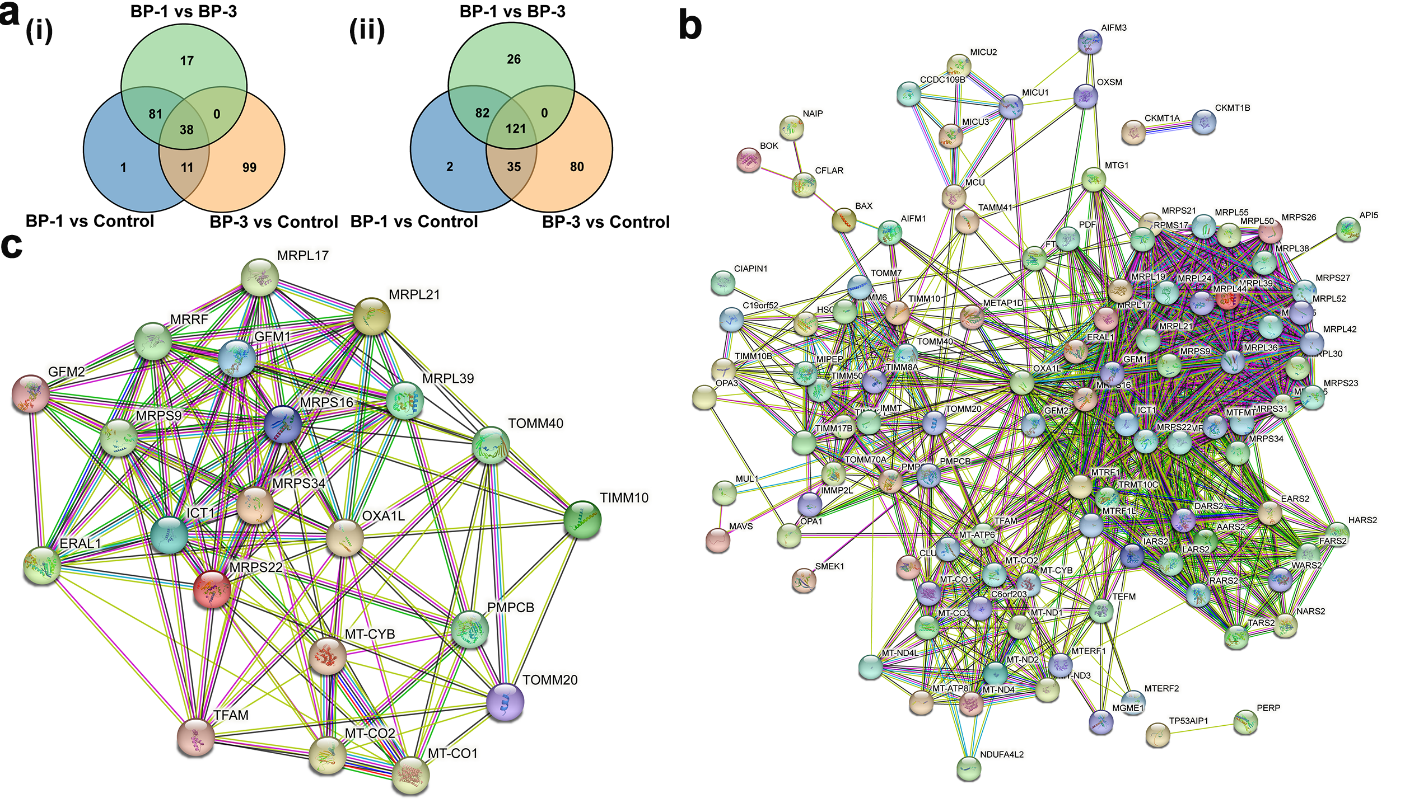
**

**Figure S6.** PPI screening of Hub genes in three comparison groups of DEGs. a: DEGs in the three comparison groups. b: PPI screening of total DEGs. c: PPI obtained by screening with degree, betweenness, and closeness averages. ((i) Up-regulated DEGs, (ii) Down-regulated DEGs)

Table S1

**Table S1**

Forward and reverse sequence of different primers.

| **Gene name** | **Sequences (5’-3’)** | **Product size** |
| --- | --- | --- |
| *β-actin*-F | CACATTGGCAATGAGCGGTTC | 145 bp |
| *β-actin*-R | AGGTCTTTGCGGATGTCACGT |  |
| *Bcl-2*-F | ATCGCCTGTGGATGACTGAGT | 135 bp |
| *Bcl-2*-R | GCCAGAGAATCAAACAGAGC |  |
| *Bax*-F | CCCGAGAGGTCTTTTCCGAG | 116 bp |
| *Bax*-R | CCCGCCCATGATGTTTTGAT |  |
| *Caspase-3*-F | TGGACTGTGGCATTGAG | 103 bp |
| *Caspase-3*-R | CCAGGTGTGGAGTA |  |

Table S2

**Table S2**

Binding energy of molecular docking

| **Ligand** | **Receptor** | **Binding energy（kcal·mol^-1^）** |
| --- | --- | --- |
| BP-1 | SOD1 | -5.301 |
|  | CAT | -8.842 |
|  | GPX1 | -5.806 |
|  | LDH-A | - 6.316 |
| BP-3 | SOD1 | -5.365 |
|  | CAT | -8.365 |
|  | GPX1 | -5.840 |
|  | LDH-A | -6.569 |
